# Perineural Dexmedetomidine Attenuates Inflammation in Rat Sciatic Nerve via the NF-κB Pathway

**DOI:** 10.3390/ijms15034049

**Published:** 2014-03-06

**Authors:** Yan Huang, Yi Lu, Lei Zhang, Jia Yan, Jue Jiang, Hong Jiang

**Affiliations:** Department of Anesthesiology, School of Medicine, Shanghai Ninth People’s Hospital Affiliated to Shanghai Jiao Tong University, Shanghai 200011, China; E-Mails: huangyan606@126.com (Y.H.); luyi077@163.com (Y.L.); weiymzhl@126.com (L.Z.); mzkyanj@163.com (J.Y.); jiangjue1136@sina.com (J.J.)

**Keywords:** dexmedetomidine, NF-κB, IL-6, TNF-α, nerve block

## Abstract

Recent studies have shown that dexmedetomidine exerts an anti-inflammatory effect by reducing serum levels of inflammatory factors, however, the up-stream mechanism is still unknown. The transcription factor NF-κB enters the nucleus and promotes the transcription of its target genes, including those encoding the pro-inflammatory cytokines IL-6 and TNF-α. In this study, we established a rat model that simulates a clinical surgical procedure to investigate the anti-inflammatory effect of perineural administration of dexmedetomidine and the underlying mechanism. Dexmedetomidine reduced the sciatic nerve levels of IL-6 and TNF-α at both the mRNA and protein level. Dexmedetomidine also inhibited the translocation of activated NF-κB to the nucleus and the binding activity of NF-κB. The anti-inflammatory effect is confirmed to be dose-dependent. Finally, pyrrolidine dithiocarbamate also reduced the levels of IL-6 and TNF-α and the activation of NF-κB. In conclusion, dexmedetomidine inhibited the nuclear translocation and binding activity of activated NF-κB, thus reducing inflammatory cytokines.

## 1. Introduction

Dexmedetomidine, a highly selective α2-adrenoceptor agonist, is widely used in clinical anesthesia, intensive care unit (ICU) management and pain treatment as a sedative agent [1–3]. Recent studies found that dexmedetomidine has an anti-inflammatory effect through reducing the serum levels of inflammatory factors, which may extend its application in the clinic [4–6]. However, the upstream mechanism by which dexmedetomidine reduces inflammatory factors levels remains largely unknown.

The NF-κB family contains five members, RelA (also known as p65), RelB, c-Rel, p105/p50, and p100/p52, which make homo- and heterodimers. NF-κB is a transcription factor that recognizes a common consensus DNA sequence and regulates a large number of target genes, especially genes involved in inflammation, injury and stress [7]. Interleukin (IL)-6 and tumor necrosis factor (TNF)-α are cytokines that play essential roles in inflammation. Studies have shown that NF-κB exists as a p65 and p50 heterodimer in the cytoplasm. Activated NF-κB enters the nucleus, where it can promote the transcription of its target genes, including the pro-inflammatory cytokines IL-6 and TNF-α. Pyrrolidine dithiocarbamate (PDTC), a selective NF-κB inhibitor and antioxidant, can inhibit the NF-κB pathway by blocking the entrance of activated NF-κB to the nucleus and the binding of NF-κB to the promoter regions of IL-6 and TNF-α [8–10].

Peripheral nerve block is a common regional anesthetic technique performed everyday throughout the world as an alternative to general anesthesia and is performed for postoperative analgesia [11–13]. Dexmedetomidine is usually injected in the peripheral nerve to prolong the duration of the peripheral nerve block as an adjuvant for local anesthetics [14–17]. In this study, perineural administration of dexmedetomidine not only blocked NF-κB translocation to the nucleus and NF-κB binding activity but also reduced IL-6 and TNF-α levels in rats. PDTC, an NF-κB inhibitor, can also reduce IL-6 and TNF-α levels by inhibiting NF-κB activity following perineural administration. In summary, dexmedetomidine can inhibit inflammation through the NF-κB pathway.

## 2. Results

### 2.1. Perineural High Doses of Dexmedetomidine Reduced the IL-6 and TNF-α Levels in the Sciatic Nerve

**Figure 1.**
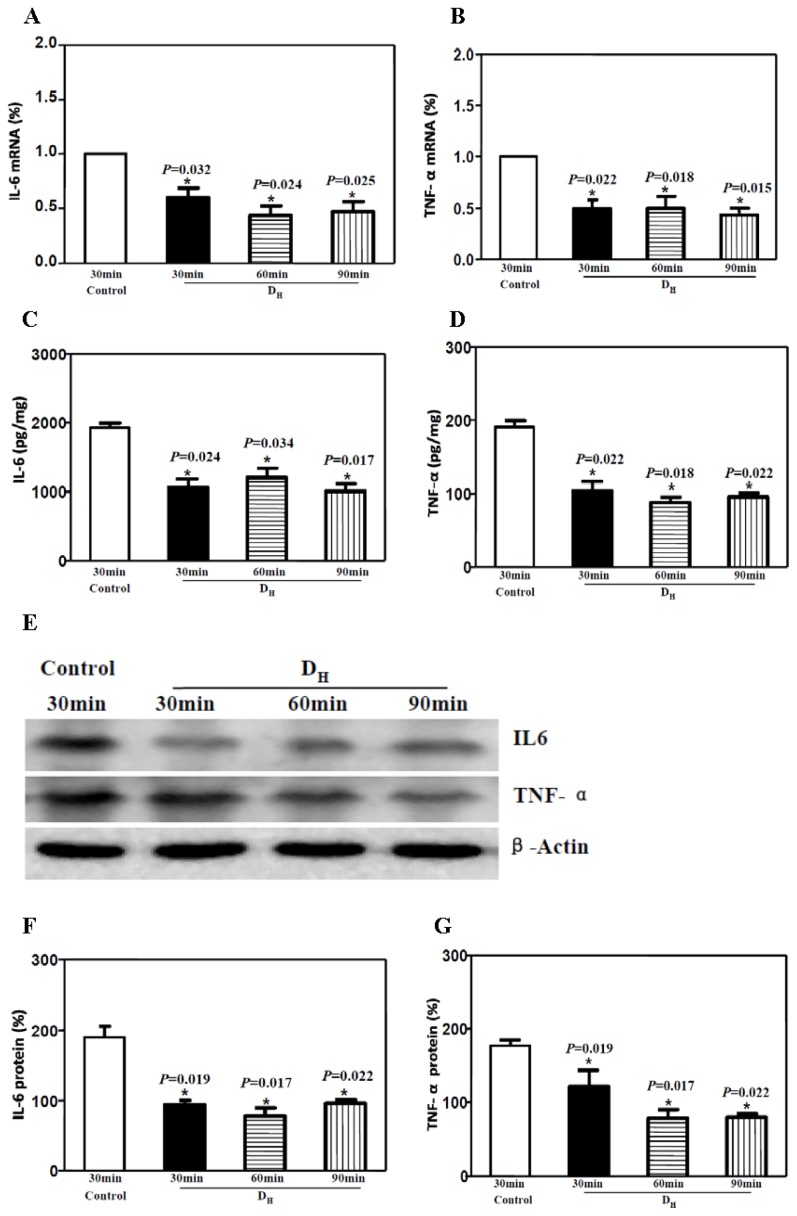
Perineural high doses (20 μg/kg) of dexmedetomidine reduced IL-6 and TNF-α levels in the sciatic nerve. (**A**,**B**) RT-PCR showed that high doses of dexmedetomidine reduced the IL-6 and TNF-α mRNA levels in the sciatic nerve; (**C**,**D**) ELISA showed that high dose of dexmedetomidine reduced the IL-6 and TNF-α protein levels in the sciatic nerve; (**E**–**G**) Western blotting showed that high dose of dexmedetomidine reduced IL-6 and TNF-α protein levels in the sciatic nerve.

Rats were anesthetized and injected perineurally with either a high dose of dexmedetomidine (D_H_, 20 μg/kg) or normal saline (C). Sciatic nerves were harvested 30, 60 and 90 min after injection. Real-time PCR revealed that dexmedetomidine reduced the mRNA levels of both IL-6 and TNF-α at all of the examined time points (Figure 1A,B). The protein levels of both IL-6 and TNF-α were significantly decreased after perineural injection of dexmedetomidine at all time points, as shown by both ELISA (Figure 1C,D) and Western blotting (Figure 1E–G). These results suggested that perineural injection of 20 μg/kg dexmedetomidine reduces the sciatic nerve levels of IL-6 and TNF-α at the level of both mRNA and protein.

### 2.2. Perineural Dexmedetomidine Decreased NF-κB Translocation to the Nucleus and Transcriptional Binding Activity

**Figure 2.**
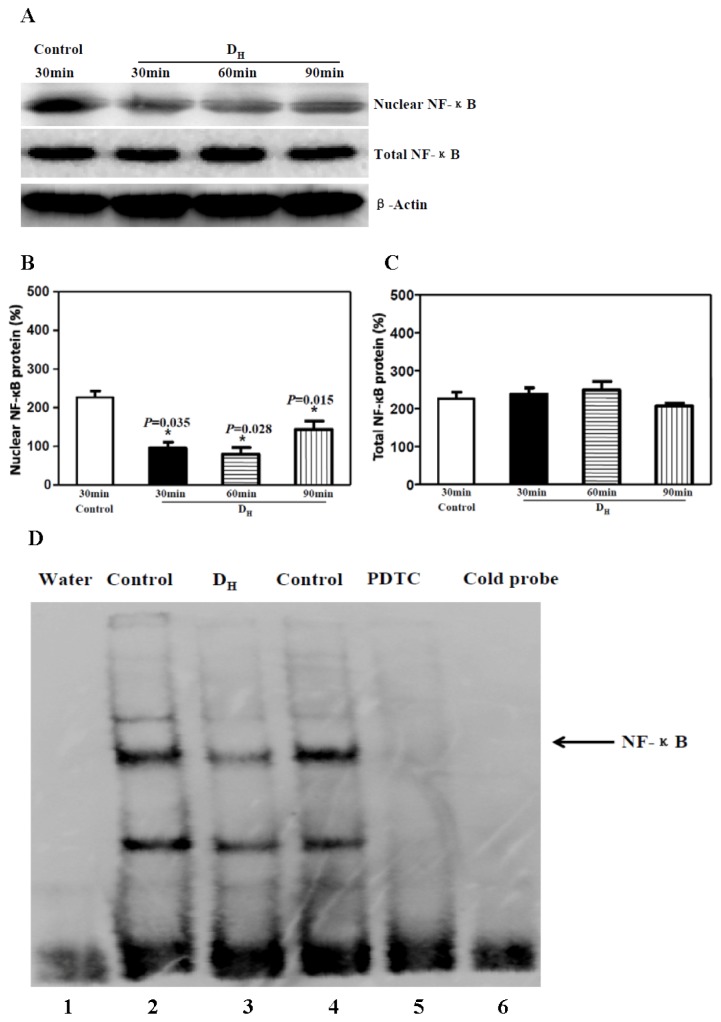
Perineural administration of 20 μg/kg dexmedetomidine decreased the translocation to the nucleus and transcriptional binding activity of NF-κB. (**A**) Western blot showed that 20 μg/kg dexmedetomidine decreased NF-κB translocation into the nucleus; (**B**) Dexmedetomidine (20 μg/kg) decreased the nuclear NF-κB protein level; (**C**) Dexmedetomidine (20 μg/kg) did not alter the total NF-κB protein level; (**D**) EMSA showed that 20 μg/kg dexmedetomidine decreased the transcriptional binding activity of NF-κB (lane 3); PDTC also decreased NF-κB transcriptional binding activity (lane 5). NF-κB, nuclear factor-kappa B; PDTC, pyrrolidine dithiocarbamate.

As previous studies have shown [16–19], activated NF-κB should translocate to the nucleus and bind to the promoter region of multiple genes, including cytokine genes, inducing the expression of cytokine mRNA and protein. Thus, we assessed the effects of perineural administration of dexmedetomidine on the nuclear levels of NF-κB in sciatic nerve tissue. The sciatic nerve tissues were subjected to Western blot analysis for the total NF-κB protein level, and the nuclear extracts were assessed for the nuclear NF-κB protein level. The Western blot of NF-κB showed that perineural administration of dexmedetomidine decreased the nuclear level of NF-κB compared with the control group at 30, 60 and 90 min (Figure 2A,B), but no significant differences were detected in the total level of NF-κB (Figure 2A,C). These findings indicate that perineural administration of dexmedetomidine may prevent NF-κB translocation into the nucleus in the sciatic nerve and thus may decrease the subsequent expression of inflammatory factors.

We then used EMSA to test the effect of dexmedetomidine on the transcriptional binding activity of NF-κB in nuclear extracts prepared from sciatic nerve tissues [20]. In Figure 2D, lane 1 is a negative control (double-distilled water), lane 2 represents the control group with perineural administration of normal saline, lane 3 represents the D_H_ group with perineural 20.0 μg/kg dexmedetomidine, and lane 6 shows the cold probe. We can clearly see that perineural administration of 20.0 μg/kg dexmedetomidine reduced the transcriptional binding activity of NF-κB compared with the control group.

### 2.3. Only High Dose Dexmedetomidine Attenuated the NF-κB Translocation to the Nucleus

**Figure 3.**
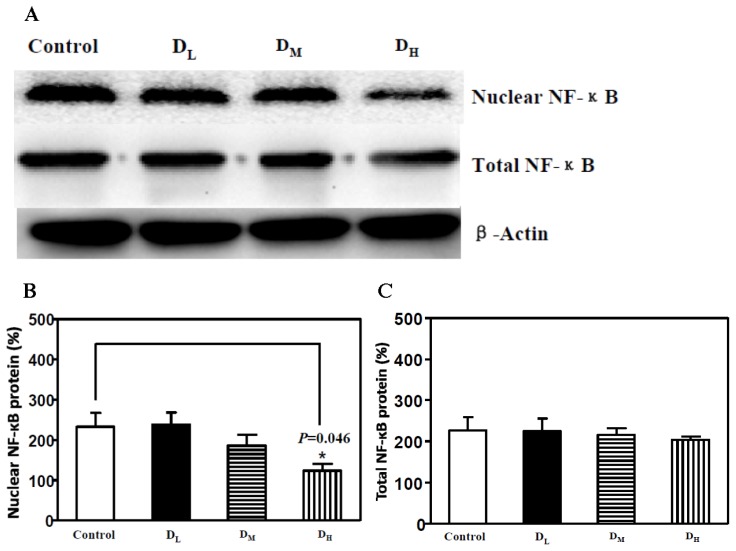
Only the high dose (20 μg/kg) of dexmedetomidine attenuated NF-κB translocation to the nucleus. (**A**) Western blotting showed that only the high dose of dexmedetomidine decreased NF-κB translocation to the nucleus; (**B**) The high dose of dexmedetomidine decreased the nuclear NF-κB protein level, but the middle doses (10 μg/kg) and low doses (5 μg/kg) did not have an effect; (**C**) Dexmedetomidine did not alter the total NF-κB protein level in any of the groups. NF-κB, nuclear factor-kappa B.

Based on the above conclusions, we further tested whether different doses of dexmedetomidine have the same effect on attenuating the translocation of NF-κB. We further tested the D_M_ and D_L_ groups, which received a middle dose and a low dose of dexmedetomidine (10.0 and 5 μg/kg, respectively). Western blotting was used to analyze the total NF-κB protein level and the nuclear NF-κB protein level. The results showed that only the high dose of dexmedetomidine (D_H_) attenuated NF-κB translocation to the nucleus, whereas the total and nuclear protein levels of NF-κB were unchanged in the D_M_ and D_L_ groups (Figure 3A–C). These results suggested that perineurally administered dexmedetomidine attenuates the inflammatory response above a certain threshold dose.

## 2.4. The Inflammation of the Sciatic Nerve Could Be Attenuated by PDTC via the NF-κB Pathway

**Figure 4.**
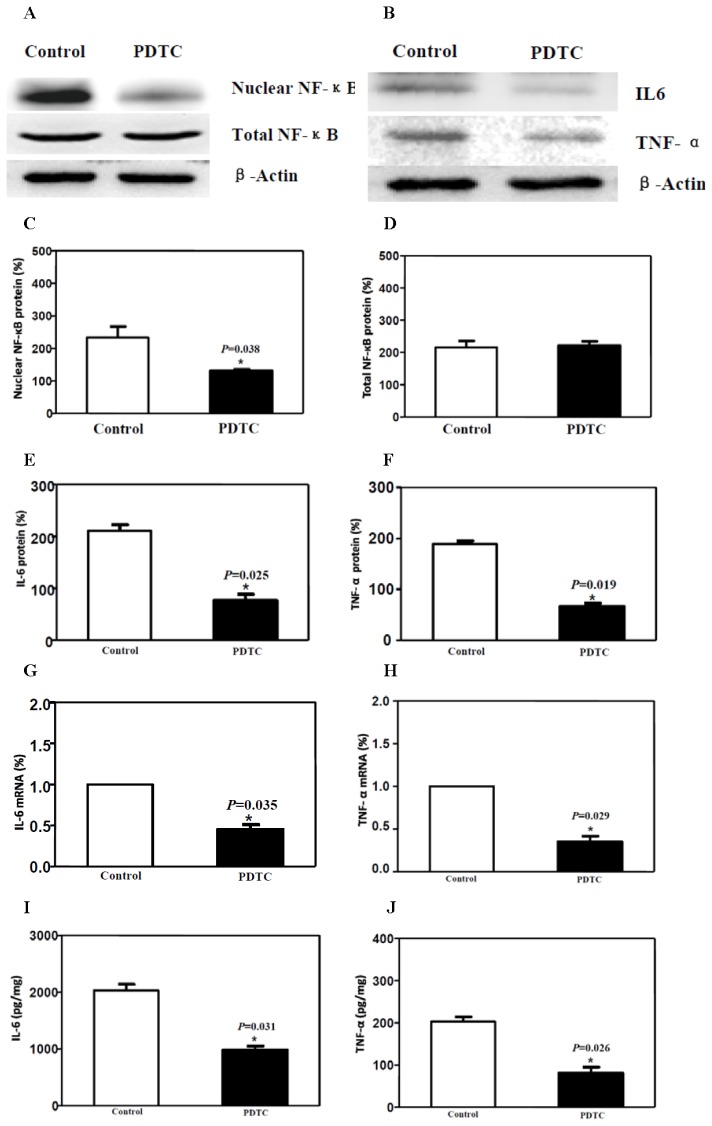
PDTC attenuated the inflammation of the sciatic nerve via the NF-κB pathway. (**A**,**C**,**D**) Western blotting showed that PDTC decreased NF-κB translocation into the nucleus; (**B**,**E**,**F**) Western blotting showed that PDTC reduced the IL-6 and TNF-α protein levels in the sciatic nerve; (**G**,**H**) RT-PCR showed that PDTC reduced the IL-6 and TNF-α mRNA levels in the sciatic nerve; (**I**,**J**) ELISA showed that PDTC reduced the IL-6 and TNF-α protein level in the sciatic nerve.

PDTC, a selective NF-κB inhibitor and antioxidant, inhibits the NF-κB pathway by blocking the translocation of activated NF-κB to the nucleus [10]. Here, we assessed whether inflammation of the sciatic nerve could be attenuated by PDTC via the NF-κB pathway in a manner similar to that of dexmedetomidine. Western blot analysis confirmed that both the translocation of NF-κB to nucleus and the downstream expression of IL-6 and TNF-α protein were down-regulated by PDTC (Figure 4A–F). RT-PCR (Figure 4G,H) and ELISA (Figure 4I,J) further confirmed the down-regulation of the inflammatory factors IL-6 and TNF-α. EMSA showed that the transcriptional binding activity of NF-κB was reduced by PDTC compared with the control group (Figure 2D, lane 5). These changes were in accordance with our results with dexmedetomidine and suggest that perineural administration of dexmedetomidine has a similar, or at least partly similar, anti-inflammatory effect as PDTC, via the NF-κB signalling pathway.

## 3. Discussion

Dexmedetomidine is widely used in clinical anesthesia as a most closely ideal sedative because of its analgesia and sedation effects without respiratory depression [18–21]. Recent studies found that dexmedetomidine has an anti-inflammatory effect by reducing the serum levels of inflammatory cytokines, however, the upstream mechanism is still unknown [4–6]. In the present study, we used a surgical procedure and perineural injection to induce a background inflammatory response (e.g., increasing IL-6 and TNF-α levels) in sciatic nerve tissue to investigate the anti-inflammatory effect of perineural administration of dexmedetomidine and the underlying mechanism. We found that, in the sciatic nerves of rats, perineural administration of 20 μg/kg dexmedetomidine reduced the level of IL-6 and TNF-α, prevented NF-κB translocation to the nucleus and decreased the transcriptional binding activity of NF-κB. These results, in accordance with perineural administration of the NF-κB pathway inhibitor PDTC, suggested that dexmedetomidine reduced inflammation by inhibiting the NF-κB signaling pathway.

Dexmedetomidine is usually intravenously administered during clinical anesthesia as a sedative agent. Currently, perineural administration of dexmedetomidine is widely used in peripheral nerve anesthesia as an adjuvant for local anesthetics to prolong the duration of a peripheral nerve block [3,17,22]. Here, we incised the skin and subcutaneous fat of rats to simulate the clinical surgical procedure. We then exposed the sciatic nerve and injected dexmedetomidine to investigate the inflammatory cytokine levels in the sciatic nerve tissue. As a result, we confirmed that perineural administration of dexmedetomidine inhibited the translocation of activated NF-κB to the nucleus and the binding activity of activated NF-κB, thus reducing the level of inflammatory cytokines. The anti-inflammatory effect of perineural administration of dexmedetomidine may extend its application in clinical anesthesia.

In a previous study [16], dexmedetomidine added to ropivacaine was shown to increase the duration of the sensory block in a dose-dependent fashion, varying from 0.5 to 20 μg/kg, in rats. Interestingly, in our study, NF-κB translocation to the nucleus was not inhibited in the D_M_ and D_L_ groups (which received dexmedetomidine doses of 10.0 and 5 μg/kg, respectively), but the D_H_ group (20 μg/kg) did show an effect. The results indicate that a dose threshold needs to be crossed before an anti-inflammatory effect occurs. Furthermore, Brummett’s study confirmed that a high dose of dexmedetomidine, up to 40 μg/kg, was considered safe for rats because the histopathological evaluation showed that nerve axon and myelin were not altered by dexmedetomidine and there was no neurotoxicity or side-effects for the rats [15]. Similarly, there was no neurotoxicity or side-effect noted at 24 h or 14 days in our study. Based on these results, a high perineural dose of dexmedetomidine may be recommended for routine use as an adjuvant for local anesthetics owing to its anti-inflammatory and analgesia-prolonging effect.

## 4. Experimental Section

All of the investigators followed the Shanghai Ninth People’s Hospital Animal Study Guidelines, and the study was approved by the Shanghai Ninth People’s Hospital Committee for the Use and Care of Animals (Shanghai, China).

For blinding, all of the drug solutions were prepared in syringes without labels by an investigator other than the one performing the perineural sciatic injection. The investigators who harvested the tissue and performed the later Western blotting, real-time PCR, ELISA and EMSA were also blinded to the study groups.

### 4.1. Animals

Forty-eight Sprague-Dawley rats, weighing 180 to 220 g, were obtained from Shanghai Ninth People’s Hospital SPF Animal Center (Shanghai, China). The rats were housed at 23 °C with a light-dark cycle and allowed free access to food and water.

### 4.2. Study Groups

The Sprague-Dawley rats were divided into four groups for further intervention. Control group rats received only normal saline. The D_H_ group received a high dose of dexmedetomidine (20.0 μg/kg), whereas the D_M_ and D_L_ groups received a middle dose and a low dose of dexmedetomidine (10.0 and 5 μg/kg, respectively).

### 4.3. Drug Preparation

An investigator who was not involved in either the perineural sciatic injection or the subsequent analysis prepared the drugs. Dexmedetomidine and normal saline were used to make final drug solutions. Each rat received a total volume of 0.2 mL for a perineural sciatic injection. The doses of dexmedetomidine were based on the individual rats’ body weight for 20.0 μg/kg (D_H_), 10.0 μg/kg (D_M_), 5.0 μg/kg (D_L_) respectively.

### 4.4. Animal Model and Perineural Injection

To investigate the anti-inflammatory effect of perineural administration of dexmedetomidine, we established a rat model of surgical-induced inflammation. Rats were anesthetized and maintained using 3.0% isoflurane. As previously described [15], an incision was made over the thigh, and the muscle and fascia were dissected to expose the sciatic nerve directly below the clear fascial covering. A total volume of 0.2 mL was injected into the perineural space below the clear fascia covering the nerve using a 30-gauge needle and tuberculin syringe. After injection, the muscle and skin of the thigh were sutured, and the isoflurane was discontinued. The animal model of surgical-induced inflammation was confirmed by increasing the inflammatory factor, such as IL-6 and TNF-α, after the surgical procedure.

### 4.5. Nuclear Extraction and Western Blotting

A nuclear extraction kit (Ab110168, Abcam, Cambridge, MA, USA) was used for the preparation of nuclear extracts from sciatic nerve tissues. For Western blotting, frozen rat sciatic nerve tissues were homogenized and the lysates were prepared in ice-cold lysis buffer. Nuclear extracts or total protein were collected and normalized for equal amounts of total protein measured by the bicinchoninic acid (BCA) method. Seventy micrograms of protein from each sample was separated on a sodium dodecyl sulfate polyacrylamide gel and transferred to PVDF membranes. The membranes were blocked with 5% nonfat milk and incubated overnight with primary anti-NF-κB antibody (1:1000; sc-109, Santa Cruz, CA, USA), anti-IL-6 antibody (1:1000; ab6672, Abcam, Cambridge, MA, USA), anti-TNF-α antibody (1:1000; AB1837P; Millipore, Billerica, MA, USA), and anti-β-actin (1:5000, Sigma, St. Louis, MO, USA) at 4 °C, followed by incubation with the suitable HRP-conjugated secondary antibody for 4 h. β-actin protein was immunodetected as the internal standard.

### 4.6. Real-Time PCR and ELISA

The levels of IL-6 and TNF-α mRNA were detected by real-time polymerase chain reaction (RT-PCR) in the sciatic nerve tissues as described [11]. RNA was isolated following the protocol of the RNeasy Mini Kit (Qiagen, Inc., Valencia, CA, USA), while concentration determined using a NanoDrop ND-1000 Spectrophotometer (Thermo Scientific, Wilmington, DE, USA). Primers of Rat IL-6 is 5′-GACTGATGTTGTTGACAGCCACTGC-3′; 5′-TAGCCACTCCTTCTGTGACTCTAACT-3′, TNF-α 5′-TTC TGT CTA CTG AAC TTC GGG GTG ATG GGT CC-3′; 5′-GTA TGA GAT AGC AAA TCG GCT GAC GGT GTG GG-3′ and Rat GAPDH 5′-CCT TCA TTG ACC TCA ACT AC-3′ ;5′-GGA AGG CCA TGC CAG TGA GC-3′. RT-PCR was carried out using the QuantiTect SYBR Green RT-PCR Kit (Qiagen). The quantity of the target mRNA was normalised against a house-keeping gene, GAPDH, which served as an internal control.

The levels of IL-6 and TNF-α proteins in the sciatic nerve were measured using ELISA kits according to the manufacturer’s instructions. ELISA kits for TNF-α and IL-6 were obtained from R & D Systems (Minneapolis, MN, USA).

### 4.7. Electrophoretic-Mobility Shift Assay (EMSA)

An electrophoretic-mobility shift assay (EMSA) kit (GS-0030, Signosis, Santa Clara, CA, USA) was used to assess the transcriptional binding activity of NF-κB as a previous study described [11]. The nuclear extract (5 mg) was incubated with 1 μL poly d(I-C), 2.0 μL 5× Binding Buffer and 1.0 μL of transcription factor (TF) probe in a 0.5 mL microcentrifuge tube at 20–23 °C for 30 min in a PCR machine. We added 1.0 μL of cold TF probe into this reaction for the cold probe control. Samples were then loaded onto a 6.5% non-denaturing polyacrylamide gel and separated at 100 V, and the proteins were transferred to a membrane at 60 V for 1 h at 4 °C. The membrane was imaged using a chemiluminescence imaging system (Bio-Rad, Hercules, CA, USA).

### 4.8. Statistics

Data are shown as mean (SD). Student’s *t*-test, one-way and two-way analysis of variance (ANOVA) were used to compare the differences among the experimental groups and the control group. *p* < 0.05 (*) was considered statistically significant. The significance testing was two-tailed. Normality tests showed that the data were normally distributed, and the GraphPad software Prism 5 (GraphPad Software Inc., San Diego, CA, USA) was used to analyze the data.

## 5. Conclusions

In conclusion, we have established a rat model that simulates a clinical surgical procedure to investigate the anti-inflammatory effect of perineural administration of dexmedetomidine and the underlying mechanism. Dexmedetomidine (20 μg/kg) inhibited the translocation of activated NF-κB to the nucleus and its binding activity, thus reducing inflammatory cytokine levels. These results suggest a potential application for dexmedetomidine as an adjuvant in peripheral nerve anesthesia. Future research should focus on finding clinical evidence to support the use of dexmedetomidine as an anti-inflammatory adjuvant for perineural administration in humans.
